# Systemic opioid regimens for postoperative pain in neonates

**DOI:** 10.1002/14651858.CD015016.pub3

**Published:** 2023-04-05

**Authors:** Mari Kinoshita, Israel Junior Borges do Nascimento, Lea Styrmisdóttir, Matteo Bruschettini

**Affiliations:** Department of PediatricsClinical Sciences Lund, Lund UniversityLundSweden; Fetal Medicine Research CenterUniversity of BarcelonaBarcelonaSpain; School of Medicine and University HospitalUniversidade Federal de Minas Gerais (UFMG)Belo HorizonteBrazil; Department of MedicineMedical College of WisconsinMilwaukeeWisconsinUSA; Faculty of MedicineLund UniversityLundSweden; Paediatrics, Department of Clinical Sciences LundLund University, Skåne University HospitalLundSweden; Cochrane Sweden, Department of Research and EducationLund University, Skåne University HospitalLundSweden

**Keywords:** Child, Child, Preschool, Humans, Infant, Infant, Newborn, Analgesia, Analgesia/methods, Analgesics, Opioid, Analgesics, Opioid/therapeutic use, Clinical Protocols, Morphine, Morphine/therapeutic use, Pain, Postoperative, Pain, Postoperative/drug therapy

## Abstract

**Background:**

Postoperative pain clinical management in neonates has always been a challenging medical issue. Worldwide, several systemic opioid regimens are available for pediatricians, neonatologists, and general practitioners to control pain in neonates undergoing surgical procedures. However, the most effective and safe regimen is still unknown in the current body of literature.

**Objectives:**

To determine the effects of different regimens of systemic opioid analgesics in neonates submitted to surgery on all‐cause mortality, pain, and significant neurodevelopmental disability. Potentially assessed regimens might include: different doses of the same opioid, different routes of administration of the same opioid, continuous infusion versus bolus administration, or 'as needed' administration versus 'as scheduled' administration.

**Search methods:**

Searches were conducted in June 2022 using the following databases: Cochrane Central Register of Controlled Trials [CENTRAL], PubMed, and CINAHL. Trial registration records were identified via CENTRAL and an independent search of the ISRCTN registry.

**Selection criteria:**

We included randomized controlled trials (RCTs), quasi‐randomized, cluster‐randomized, and cross‐over controlled trials evaluating systemic opioid regimens' effects on postoperative pain in neonates (pre‐term or full‐term). We considered suitable for inclusion: I) studies evaluating different doses of the same opioid; 2) studies evaluating different routes of administration of the same opioid; 3) studies evaluating the effectiveness of continuous infusion versus bolus infusion; and 4) studies establishing an assessment of an 'as needed' administration versus 'as scheduled' administration.

**Data collection and analysis:**

According to Cochrane methods, two investigators independently screened retrieved records, extracted data, and appraised the risk of bias. We stratified meta‐analysis by the type of intervention: studies evaluating the use of opioids for postoperative pain in neonates through continuous infusion versus bolus infusion and studies assessing the 'as needed' administration versus 'as scheduled' administration. We used the fixed‐effect model with risk ratio (RR) for dichotomous data and mean difference (MD), standardized mean difference (SMD), median, and interquartile range (IQR) for continuous data. Finally, we used the GRADEpro approach for primary outcomes to evaluate the quality of the evidence across included studies.

**Main results:**

In this review, we included seven randomized controlled clinical trials (504 infants) from 1996 to 2020. We identified no studies comparing different doses of the same opioid, or different routes. The administration of continuous opioid infusion versus bolus administration of opioids was evaluated in six studies, while one study compared 'as needed' versus 'as scheduled' administration of morphine given by parents or nurses. Overall, the effectiveness of continuous infusion of opioids over bolus infusion as measured by the visual analog scale (MD 0.00, 95% confidence interval (CI) ‐0.23 to 0.23; 133 participants, 2 studies; I² = 0); or using the COMFORT scale (MD ‐0.07, 95% CI ‐0.89 to 0.75; 133 participants, 2 studies; I² = 0), remains unclear due to study designs' limitations, such as the unclear risk of attrition, reporting bias, and imprecision among reported results (very low certainty of the evidence).  None of the included studies reported data on other clinically important outcomes such as all‐cause mortality rate during hospitalization, major neurodevelopmental disability, the incidence of severe retinopathy of prematurity or intraventricular hemorrhage, and cognitive‐ and educational‐related outcomes.

**Authors' conclusions:**

Limited evidence is available on continuous infusion compared to intermittent boluses of systemic opioids. We are uncertain whether continuous opioid infusion reduces pain compared with intermittent opioid boluses; none of the studies reported the other primary outcomes of this review, i.e. all‐cause mortality during initial hospitalization, significant neurodevelopmental disability, or cognitive and educational outcomes among children older than five years old. Only one small study reported on morphine infusion with parent‐ or nurse‐controlled analgesia.

## Plain language summary

**How effective and safe are systemic opioids for postoperative pain control and management in neonates?**

**Review Question**

How effective and safe are systemic opioids for reducing newborn babies' pain after surgery?

**Background**

Neonates (babies in the first four weeks after birth) may undergo surgery (operations) or surgical procedures. Like adults, babies experience pain, and this pain must be managed (reduced) after surgery. Opioids are pain‐relieving medications. Examples of opioids are codeine and morphine. Opioids work by interacting with opioid receptors in the body and reducing feelings of pain.

Opioids affect the whole body system and this is why this review refers to them as systemic opioids. Opioids can be given to babies in a few ways, by different routes. One route is by using a needle injected into a vein; this is called parenteral drug administration. Another way (or route) is to place a medication in the baby's mouth, under the tongue or with a tube. These types of drug delivery are called enteral administration. Opioids, like most drugs, can be given at different strengths (dosages). Opioids can be given continuously (without stopping), or on and off over a period of time (intermittently).

All of these things together, how the opioid is given to the baby, how often the opioid is given, and the strength of the opioid, create what is called a drug regimen.

This review aims to evaluate how different opioid regimens affect babies.

**Key results**

This review included seven studies involving 504 babies. We identified no studies comparing different doses of the same opioid. We identified no studies comparing different routes to delivery of opioids. Six studies compared continuous opioid administration versus intermittent opioid administration. One study assessed the use of continuous morphine infusion compared with a parent‐ or nurse‐controlled administration.

Based on the studies we found that we were unable to determine whether continuous or intermittent opioid regimens are better for controlling babies' pain. Since we did not find studies comparing different dosages of opioids, we do not know which dosage is better for reducing babies' pain. Since we did not find studies comparing different routes of opioid administration, we do not know if parenteral is better than enteral for reducing babies' pain. Considering the body of literature evaluated, the effectiveness of continuous systemic opioid infusion compared with intermittent systemic opioid administration is still undetermined. We are uncertain about the effectiveness of continuous systemic opioid administration and intermittent opioid administration in reducing the pain.  We searched for studies that were available up to 10 June 2022.

## Summary of findings

**Summary of findings 1:** Continuous infusion compared to bolus administration for postoperative pain in neonates

**Continuous infusion compared to bolus administration for postoperative pain in neonates**						
**Patient or population:** postoperative pain in neonates **Setting:** neonatal intensive care units **Intervention:** continuous infusion **Comparison:** bolus administration						
**Outcomes**	Anticipated absolute effects^*^ (95% CI)		**Relative effect (95% CI)**	**№ of participants (studies)**	**Certainty of the evidence (GRADE)**	**Comments**
	**Risk with bolus administration**	**Risk with continuous infusion**				
Pain assessed with visual analogue scale (VAS) during the administration of selected drugs (neonates from 0 to 4 weeks) VAS scale ranges from 0 to 10 (worst)	The mean pain assessed with VAS was 1.3	The mean pain assessed with VAS was 1.3	MD 0 (0.23 lower to 0.23 higher)	133 (2 RCTs)	⊕⊝⊝⊝ Very low ^a,b^	We are uncertain whether opioid continuous infusion reduces pain assessed with a visual analogue scale (VAS) compared with bolus administration due to imprecision of the estimate and limitations in study design.
Pain assessed with COMFORT scale during the administration of selected drugs (neonates from 0 to 4 weeks) COMFORT scale ranges from 6 to 30 (worst)	The pain assessed with COMFORT ranged from 12.8 to 17.3	The pain assessed with COMFORT ranged from 12.6 to 17.4	MD 0.07 lower (0.89 lower to 0.75 higher)	133 (2 RCTs)	⊕⊝⊝⊝ Very low ^a,b^	We are uncertain whether opioid continuous infusion reduces pain assessed with the COMFORT scale compared with bolus administration due to imprecision of the estimate and limitations in study design.
All‐cause mortality during initial hospitalization ‐ not reported	‐	‐	‐	‐	‐	This outcome was not reported
Major neurodevelopmental disability in children aged 18 to 24 months or three to five years old ‐ not reported	‐	‐	‐	‐	‐	This outcome was not reported
Cognitive and educational outcomes in children more than five years old ‐ not reported	‐	‐	‐	‐	‐	This outcome was not reported
Severe (defined as stage 3 or greater) retinopathy of prematurity ‐ not reported	‐	‐	‐	‐	‐	This outcome was not reported
Severe (grade 3 or greater) intraventricular hemorrhage (IVH) on cranial ultrasound ‐ not reported	‐	‐	‐	‐	‐	This outcome was not reported
*The risk in the intervention group (and its 95% confidence interval) is based on the assumed risk in the comparison group and the **relative effect** of the intervention (and its 95% CI). **CI:** confidence interval; **OR:** odds ratio; **RR:** risk ratio;						
**GRADE Working Group grades of evidence** **High certainty:** we are very confident that the true effect lies close to that of the estimate of the effect. **Moderate certainty:** we are moderately confident in the effect estimate: the true effect is likely to be close to the estimate of the effect, but there is a possibility that it is substantially different. **Low certainty:** our confidence in the effect estimate is limited: the true effect may be substantially different from the estimate of the effect. **Very low certainty:** we have very little confidence in the effect estimate: the true effect is likely to be substantially different from the estimate of effect.						

VAS: visual analogue scale IVH: intraventricular hemorrhage ^a^ Downgraded one level for risk of bias in some included trials: unclear risk of attrition and reporting bias ^b^ Downgraded two levels for serious imprecision of effect estimates (wide 95% CI around estimate consistent with substantial harm or benefit)

**Summary of findings 2:** 'As needed' administration (e.g. based on pain scales) versus 'as scheduled' administration (e.g. a predefined time interval)

**'As needed' administration (e.g. based on pain scales) versus 'as scheduled' administration (e.g. a predefined time interval)**						
**Patient or population:** postoperative pain in neonates **Setting:** neonatal intensive care units **Intervention:** 'as needed' administration (e.g. based on pain scales) **Comparison:** 'as scheduled' administration (e.g. a predefined time interval)						
**Outcomes**	Anticipated absolute effects^*^ (95% CI)		**Relative effect (95% CI)**	**№ of participants (studies)**	**Certainty of the evidence (GRADE)**	**Comments**
	**Risk with 'as scheduled' administration**	**Risk with 'as needed' administration**				
Pain assessed with any of the prespecified scales, during the administration of selected drugs (neonates from 0 to 4 weeks)	‐	‐	‐	‐	‐	This outcome was not reported.
All‐cause mortality during initial hospitalization ‐ not reported	‐	‐	‐	‐	‐	This outcome was not reported.
Major neurodevelopmental disability in children aged 18 to 24 months or three to five years old ‐ not reported	‐	‐	‐	‐	‐	This outcome was not reported.
Cognitive and educational outcomes in children more than five years old ‐ not reported	‐	‐	‐	‐	‐	This outcome was not reported.
Severe (defined as stage 3 or greater) retinopathy of prematurity ‐ not reported	‐	‐	‐	‐	‐	This outcome was not reported.
Severe (grade 3 or greater) intraventricular hemorrhage (IVH) on cranial ultrasound ‐ not reported	‐	‐	‐	‐	‐	This outcome was not reported.
*The risk in the intervention group (and its 95% confidence interval) is based on the assumed risk in the comparison group and the **relative effect** of the intervention (and its 95% CI). **CI:** confidence interval; **OR:** odds ratio; **RR:** risk ratio;						
**GRADE Working Group grades of evidence** **High certainty:** we are very confident that the true effect lies close to that of the estimate of the effect. **Moderate certainty:** we are moderately confident in the effect estimate: the true effect is likely to be close to the estimate of the effect, but there is a possibility that it is substantially different. **Low certainty:** our confidence in the effect estimate is limited: the true effect may be substantially different from the estimate of the effect. **Very low certainty:** we have very little confidence in the effect estimate: the true effect is likely to be substantially different from the estimate of effect.						

IVH: intraventricular hemorrhage

## Background

### Description of the condition

Newborn infants undergo surgeries for treatment of congenital abnormalities and neonatal morbidities and are managed in the neonatal intensive care unit (NICU) thereafter. The clinical spectrum of these abnormalities ranges from conditions such as diaphragmatic hernia and gastroschisis, which require surgical repair immediately or relatively soon after birth, to conditions such as congenital heart disease and hypertrophic pyloric stenosis that can wait several weeks before being treated. Neonatal morbidities include complications often due to prematurity, such as necrotizing enterocolitis, spontaneous intestinal perforation, and retinopathy of prematurity, which require surgical treatment. Such surgical interventions result in acute pain during and after surgery, and also easily lead to chronic pain due to hyperalgesia during a vital period of complex brain development (Fitzgerald 1989).

Neonatal pain might affect neuropsychological development in the long term. Therefore, it is important to accurately identify and appropriately manage pain. However, major gaps in knowledge exist regarding both objective assessment of pain, the most effective way to prevent and relieve pain, as well as the long‐term effects of drug therapy. Systematic evaluation of pain has increased the awareness of treating pain, but pain assessment continues to pose a challenge (Olsson 2021). Although there are many validated scales for the assessment of both acute and continuous pain, a fully reliable and objective assessment method is still lacking (Eriksson 2019; Olsson 2021).

A recent review of pediatric perioperative controlled trials published between 2008 and 2018 reported that outcomes related to patient comfort, including pain management, were the most frequent across age groups beyond infancy, while clinical variables such as cardiorespiratory or medication‐related adverse events were the most common outcome for neonates and infants under 60 weeks of age (Muhly 2020). The review also pointed out that the youngest age group of neonates and infants under 60 weeks of age were significantly under‐represented in perioperative trials. This could be due to the higher perioperative risk of morbidity and mortality in neonates compared to older children (Kuan 2020), as well as to neonatal pharmacokinetics, which is not yet well characterized (Euteneuer 2020). The present reality is that optimal pain management in newborns is yet to be achieved, with further primary studies and updated systematic reviews needed for this unique age group.

### Description of the intervention

Morphine, fentanyl, and remifentanil are the opioids most often used during neonatal intensive care, whereas the fentanyl derivatives alfentanil and sufentanil are less frequently used. These opioids have varying pharmacokinetic (PK) and pharmacodynamic (PD) profiles and should optimally be administered in an individualized way, according to the need, clinical state, and expected course of the hospitalization. Fentanyl and remifentanil are administered intravenously in very sick infants, whereas morphine can be administered by both intravenous and oral routes. Morphine has the longest duration of onset, half‐life, and elimination time, followed by fentanyl and remifentanil (Thigpen 2019; Van Gonge 2018; Ziesenitz 2018). Remifentanil is a short‐acting opioid with ultra‐rapid onset and a very fast elimination profile, thus very suitable for rapid painful procedures such as endotracheal intubation (McPherson 2018). Pharmacodynamic studies on opioids report hypotension as the most common adverse effect (Thigpen 2019). Several larger studies have questioned the effect of opioids and reported on negative outcomes (Anand 2004; Hall 2005; Simons 2003). Accumulating data report on the negative impact on the structure and function of the developing brain, including neuronal apoptosis (McPherson 2015; Sanders 2013; Zwicker 2016).

### How the intervention might work

Opioids have been commonly used in postoperative management after major procedures (such as to correct cardiac or other thoracoabdominal abnormalities, and otorhinolaryngological surgeries or neurosurgeries), particularly among preterm infants (Van Dijk 2001). Their analgesic function is related to interaction with the mu, kappa, and delta receptors present in the entire central nervous system which, as a final outcome, decrease neuronal excitability and reduce neurotransmission of nociceptive impulses (Trescot 2008). The overall efficacy of opioids administered directly to the central compartment is evident even when administered at low doses. However, in the case of peripheral administration in post‐surgery, post‐trauma or inflammatory state situations, their effectiveness is not as reliable. In recent years, recommendations on time‐scheduled opioid‐dosing protocols and pain‐contingent ('as needed') control have become more common (American Academy of Pediatrics 2016). For neonates during the postoperative period, it is thought that continuous administration of opioids results in steadier serum concentration of the active metabolite, establishing better pain relief, fewer adverse effects and side effects, reduced augmentation of pain behaviors and decreased risk of abstinence syndrome.

As far as routes of administration are concerned, several possibilities can be listed. Oral administration may be difficult immediately after the surgery due to the consciousness of the infant as well as the condition of the gastrointestinal system, which is affected by administered drugs and by the surgery itself. Potential physical‐chemical interaction with milk and other frequently used medications during hospitalization (such as antibiotics) may also need to be considered (O'Brien 2019; Papai 2010). Likewise, intramuscular and subcutaneous injections are uncommon methods of opioid delivery in neonates, due to limited muscle mass, impact on skeletal muscle vascularization, and increased discomfort generated by these routes of administration (Costa 2013; Strolin 2003). Conversely, intravenous administration of opioids is most often the preferred route of administration, particularly among critically ill infants (WHO 2012). Close monitoring should be undertaken in order to prevent excess administration of total fluids to the neonate: a regular intravenous fluid infusion rate can be as low as 10 mL per hour for full‐term neonates and as low as 2 mL per hour for extremely preterm infants.

Morphine, one of the most used candidates in this category and a first‐line opioid, is typically administered through a continuous intravenous infusion, with a dose ranging from 1 to 30 mcg/kg per hour, until no more improvement in pain control is observed, indicating a dose appropriate to the individual’s current need (Anand 2004; Balda 2019). Interestingly, morphine starts working as an analgesic five minutes after the start of administration and reaches a peak effect in 15 minutes. Alternatively, an intermittent dose might be offered to the neonate, at 0.05 to 0.20 mg/kg per dose every four to six hours, preferably intravenously. Fentanyl, which begins its onset of action two to three minutes after injection, also can be given intermittently (at 0.3 to 4.0 mcg/kg per dose every two to four hours, intravenously) or as a continuous infusion (with a starting dose of slow 0.3 mcg/kg per hour, reaching a maximum dose of 5.0 mcg/kg per hour) (Anand 2004; Balda 2019). Similarly, tramadol is typically given at an increasing dose pattern (frequently administered as an intermittent medication at the dose of 5 mg/kg per day divided every 6 or 8 hours, intravenously or orally, or continuously at the dose of 0.10 to 0.25 mg/kg per hour) (Anand 2004; Balda 2019). In spite of many alternatives for pain control among neonates, the best dose regimen, route of administration and most appropriate opiate for neonates post‐surgery is still uncertain, mainly due to the physiologic and metabolic immaturity of the neonate and the potential risk of toxicity.

### Why it is important to do this review

Based on previous systematic reviews (Cochrane Reviews and non‐Cochrane reviews), the American Academy of Pediatrics highlights the conflicting findings and lack of findings published in recent years associated with the use of opioids for analgesia in neonates (American Academy of Pediatrics 2016). Some particular populations have already been widely evaluated for the use of opioids, such as mechanically ventilated neonates (Bellù 2021), and those requiring non‐emergency intubation (Ayed 2017). The assessment of the contemporary practice of analgesic and sedative procedures is of utmost importance, especially for infants in substantial pain during the postoperative period. An ongoing Cochrane Review of opioids compared to placebo or no drug, to oral sugar solution or non‐pharmacological intervention, or to other analgesics or sedatives is under preparation (Kinoshita 2021). In this review, we assess different regimens to administer systemic opioids for postoperative pain in neonates.

## Objectives

To determine the effects of different regimens of systemic opioid analgesics in neonates (term or preterm) undergoing surgery, on mortality, pain and major neurodevelopmental disability. These different regimens may include: different doses of the same opioid; different routes of administration of the same opioid; continuous infusion versus bolus administration; or 'as needed' administration versus 'as scheduled' administration.

## Methods

### Criteria for considering studies for this review

#### Types of studies

We included prospective randomized controlled trials (RCTs), quasi‐RCTs, cluster‐RCTs, and cross‐over RCTs.

#### Types of participants

We included preterm and term infants of a postmenstrual age (PMA) up to 46 weeks and 0 days, irrespective of their gestational age at birth, receiving opioids following neonatal surgery where the surgery was performed in the operating room under general anesthesia (e.g. hernia repair surgery) or in the neonatal ward for minor surgery (e.g. patent ductus arteriosus ligation, surgery for retinopathy of prematurity, positioning of surgical drainage for air leak, thoracocentesis, placement of reservoir, or peritoneal dialysis for acute kidney failure).

We excluded:

- infants receiving opioids during mechanical ventilation for respiratory morbidity;
- infants receiving opioids pre‐intubation;
- infants receiving opioids for procedural pain;
- infants treated for neonatal abstinence syndrome; and
- infants undergoing hemodialysis.

#### Types of interventions

We included studies on any opioids (e.g. morphine, diamorphine, fentanyl, alfentanil, sufentanil, pethidine, meperidine, codeine) following neonatal surgery. The following acceptable comparisons were included.

- Comparison 1: different doses of the same opioid
- Comparison 2: different routes of administration of the same opioid (e.g. enteral versus parenteral)
- Comparison 3: continuous infusion versus bolus administration of the same opioid
- Comparison 4: 'as needed' administration (e.g. based on pain scales) versus 'as scheduled' administration of the same opioid (e.g. a predefined time interval)

We included any systemic route of administration (e.g. enteral and intravenous).

We excluded spinal administration (i.e. intrathecal, epidural, caudal), intraosseous infusion, nerve blocks or wound infusions.

We included studies where the interventions were started during surgery, if their administration was continued postoperatively.

Studies comparing opioids to other interventions were included in the ongoing Cochrane Review, 'Systemic opioids versus other analgesics and sedatives for postoperative pain in neonates' (Kinoshita 2021).

#### Types of outcome measures

We focused on outcomes associated with pain assessment or management, neurological and cognitive functions, as well as other clinically relevant outcomes.

##### Primary outcomes

- Pain assessed with validated methods during the administration of selected drugs. The following scales, developed to assess pain, fulfill validity and reliability criteria for newborn infants (term and preterm on mechanical ventilation for any respiratory disease) when critically reviewed (Giordano 2019) were, as follows: Neonatal Infant Pain Scale (NIPS) (Lawrence 1983); Premature Infant Pain Profile (PIPP) (Stevens 1996); COMFORTneo (Van Dijk 2009); Neonatal Pain, Agitation and Sedation Scale (N‐PASS) (Hummel 2008), as well as Visual Analogue Scale (VAS).
- All‐cause mortality during initial hospitalization
- Major neurodevelopmental disability: cerebral palsy, developmental delay (Bayley Scales of Infant Development ‐ Mental Development Index Edition II (BSID‐MDI‐II; Bayley 1993), Bayley Scales of Infant and Toddler Development ‐ Edition III Cognitive Scale (BSITD‐III) (Bayley 2005)), or Griffiths Mental Development Scale ‐ General Cognitive Index (GCI) (Griffiths 1954; Griffiths 1970), assessment greater than two standard deviations (SDs) below the mean), intellectual impairment (intelligence quotient (IQ) greater than two SDs below the mean), blindness (vision less than 6/60 in both eyes), or sensorineural deafness requiring amplification (Jacobs 2013). We planned to separately assess data on children aged 18 to 24 months and aged three to five years.
- Cognitive and educational outcomes in children older than five years old

##### Secondary outcomes

- All‐cause neonatal mortality (death until postnatal day 28)
- Episodes of bradycardia defined as a fall in heart rate of more than 30% below the baseline or less than 100 beats per minute for 10 seconds or longer
- Hypotension requiring medical therapy (vasopressors or fluid boluses)
- Retinopathy of prematurity (ROP) in infants examined (all stages (stage 1 or greater) and severe (defined as stage 3 or greater)) (ICCROP 2005)
- Intraventricular hemorrhage (IVH; all (grade 1 or 2) or severe (grade 3 or greater) on cranial ultrasound, as per Papile classification (Papile 1978)
- Periventricular leukomalacia (PVL) (any grade (Grade 1 or greater), on basis of ultrasound or magnetic resonance imaging (De Vries 1992)
- Necrotizing enterocolitis (NEC) (modified Bell stage 2/3; Walsh 1986)
- Bronchopulmonary dysplasia/chronic lung disease:
  - 28 days (NIH 1979)
  - 36 weeks' postmenstrual age (Jobe 2001)
  - physiological definition (Walsh 2004)
- Constipation defined as a delay in defecation sufficient to cause significant distress to the infant
- Focal gastrointestinal perforation
- Duration of mechanical ventilation (days)
- Number of infants with mechanical ventilation longer than 24 hours
- Duration of oxygen supplementation (days)
- Hospital stay (days)
- Time to full enteral feeding (days)
- Cost of neonatal care

### Search methods for identification of studies

Search strategies were developed by an information specialist and peer‐reviewed by another. Database and trial registry searches were conducted without date, language, or publication type limits.

#### Electronic searches

We searched the following databases on 10 June 2022:

- Cochrane Central Register of Controlled Trials (CENTRAL 2022, Issue 6) in the Cochrane Library via Wiley;
- PubMed (1966 to 10 June 2022);
- CINAHL (1982 to 10 June 2022) via EbscoHost.

We used Cochrane Neonatal's search strategy for neonates and a methodological filter for randomized controlled trials. Search strategies are provided in Appendix 1.

Trial registration records from the World Health Organization’s International Clinical Trials Registry Platform (ICTRP) (www.who.int/ictrp/search/en/), and the United States' National Library of Medicine’s ClinicalTrials.gov (clinicaltrials.gov), were identified via Cochrane CENTRAL. We searched the ISRCTN registry (isrctn.com) independently.

#### Searching other resources

We also reviewed the reference lists of the included studies for studies not located in the database search. We searched for errata or retractions for included studies published in full text on PubMed (www.ncbi.nlm.nih.gov/pubmed).

### Data collection and analysis

We collected information regarding the method of randomization, blinding, intervention, stratification, and whether the trial was single or multicenter for each included study. We noted information regarding trial participants including birth weight, gestational age, number of participants, modality of administration and dose of opioids. We analyzed the clinical outcomes noted above in Types of outcome measures.

#### Selection of studies

Initial search results were analyzed using Known Assessments and RCT Classifier segments of Cochrane’s Screen4Me; remaining references were screened by the author. Detailed information regarding evaluations of the Screen4Me components can be found in the following publications: Marshall 2018; Noel‐Storr 2020; Noel‐Storr 2021; Thomas 2020.

We included all randomized, quasi‐randomized, cluster‐randomized and cross‐over controlled trials fulfilling our inclusion criteria. Two review authors (IJBN, LS) independently reviewed the results of the search and selected studies for inclusion. We resolved any disagreements through discussion or, when necessary, by involving a third author.

We recorded the selection process in sufficient detail to complete a PRISMA flow diagram and 'Characteristics of excluded studies' table (Moher 2009).

#### Data extraction and management

Two review authors (MK, LS) independently extracted data using a data extraction form integrated with a modified version of the Cochrane Effective Practice and Organization of Care Group data collection checklist (Cochrane EPOC Group 2017). We piloted the form within the review team using a sample of included studies.

We extracted these characteristics from each included study:

- administrative details: study author(s); published or unpublished; year of publication; year in which study was conducted; presence of vested interest; details of other relevant papers cited;
- study: study design; type, duration, and completeness of follow‐up (e.g. greater than 80%); country and location of study; informed consent; ethics approval;
- participants: sex, birth weight, gestational age, number of participants;
- interventions: initiation, dose, and duration of administration;
- outcomes as mentioned above under Types of outcome measures.

We resolved any disagreements through discussion. We described ongoing studies identified by our search, when available, detailing the primary author, research question(s), methods, and outcome measures, together with an estimate of the reporting date and reported them in the 'Characteristics of included studies' table.

If any queries arose (e.g. discrepancies in the definitions of the outcomes in the trials and under 'Types of outcome measures'), or in cases for which additional data were required, we contacted study investigators/authors for clarification. Two review authors (MK, IJBN) used Cochrane statistical software for data entry (Review Manager 2020). We replaced any standard error of the mean (SEM) by the corresponding SD.

#### Assessment of risk of bias in included studies

Two review authors (MK, LS) independently assessed the risk of bias (low, high, or unclear) of all included trials, using the Cochrane Risk of bias tool for the following domains (Higgins 2011).

- Sequence generation (selection bias)
- Allocation concealment (selection bias)
- Blinding of participants and personnel (performance bias)
- Blinding of outcome assessment (detection bias)
- Incomplete outcome data (attrition bias)
- Selective reporting (reporting bias)
- Any other bias

We resolved any disagreements through discussion or by consulting a third author (IJBN). See Appendix 2 for a more detailed description of risk of bias for each domain.

#### Measures of treatment effect

We performed the statistical analyses using Review Manager 5 software (Review Manager 2020). We summarized the data in a meta‐analysis if they were sufficiently homogeneous, both clinically and statistically.

##### Dichotomous data

For dichotomous data, we presented results using risk ratios (RR) and risk differences (RD) with 95% confidence intervals (CIs). We calculated the number needed to treat for an additional beneficial outcome (NNTB), or number needed to treat for an additional harmful outcome (NNTH) with 95% CIs if there was a statistically significant reduction (or increase) in RD.

##### Continuous data

For continuous data, we used the mean difference (MD) when outcomes were measured in the same way between trials. We used the standardized mean difference (SMD) to combine trials that measured the same outcome but used different methods. Where trials reported continuous data as a median and interquartile range (IQR) and data passed the test of skewness, we converted the median to a mean and estimated the standard deviation as IQR/1.35.

#### Unit of analysis issues

The unit of analysis was the participating infant in individually randomized trials, and an infant was considered only once in the analysis. The participating neonatal unit or section of a neonatal unit or hospital were the units of analysis in cluster‐randomized trials. We planned to analyze them using an estimate of the intracluster correlation coefficient (ICC) derived from the trial (if possible), or from a similar trial or from a study with a similar population as described in Section 16.3.6 of the *Cochrane Handbook for Systematic Reviews of Interventions* (Higgins 2021). If we used ICCs from a similar trial or from a study with a similar population, we reported this and conducted a sensitivity analysis to investigate the effect of variation in the ICC.

We acknowledged any possible heterogeneity in the randomization unit and performed a sensitivity analysis to investigate possible effects of the randomization unit.

#### Dealing with missing data

Where feasible, we carried out analysis on an intention‐to‐treat basis for all outcomes. Whenever possible, we analyzed all participants in the treatment group to which they were randomized, regardless of the actual treatment received. When we identified important missing data (in the outcomes) or unclear data, we requested the missing data by contacting the original investigators. We made explicit the assumptions of any methods used to deal with missing data. We performed sensitivity analyses to assess how sensitive results were to reasonable changes in the undertaken assumptions. We addressed the potential impact of missing data on the findings of the review in the ’Discussion’ section.

#### Assessment of heterogeneity

We estimated the treatment effects of individual trials and examined heterogeneity among trials by inspecting the forest plots and quantifying the impact of heterogeneity using the I^2^ statistic. We graded the degree of heterogeneity as:

- less than 25%: no heterogeneity;
- 25% to 49%: low heterogeneity;
- 50% to 75%: moderate heterogeneity;
- more than 75%: substantial heterogeneity.

When we noted significant statistical heterogeneity (I^2^ > 50%), we explored the possible causes (e.g. differences in study quality, participants, intervention regimens, or outcome assessments).

#### Assessment of reporting biases

We intended to conduct a comprehensive search for eligible studies and were alerted for duplication of data. We planned to assess possible publication bias by inspection of a funnel plot. If we had uncovered reporting bias that could, in the opinion of the review authors, introduce serious bias, we planned to conduct a sensitivity analysis to determine the effect of including and excluding these studies in the analysis.

#### Data synthesis

As we identified multiple studies that were considered to be sufficiently similar, we performed meta‐analysis using Review Manager 5 (Review Manager 2020). For categorical outcomes, we calculated the typical estimates of RR and RD, each with its 95% CI. For continuous outcomes, we calculated the MD (or the SMD), each with its 95% CI. We used a fixed‐effect model to combine data where it was reasonable to assume that studies were estimating the same underlying treatment effect. When we judged meta‐analysis to be inappropriate, we analyzed and interpreted individual trials separately. When there was evidence of clinical heterogeneity, we tried to explain this based on the different study characteristics and subgroup analyses.

#### Subgroup analysis and investigation of heterogeneity

We explored statistical heterogeneity in the outcomes by visually inspecting the forest plots and by removing the outlying studies in a sensitivity analysis (Higgins 2020). Where statistical heterogeneity was moderate or substantial, we interpreted the results of the meta‐analyses accordingly; and we downgraded the certainty of evidence in the Summary of findings tables, according to the GRADE recommendations.

We considered the following groups for subgroup analysis where data were available.

- Gestational age (GA): term; moderately preterm (32 to 36 weeks' GA); very preterm (less than 32 weeks' GA)
- Duration of opioids administration: up to 72 hours after surgery; beyond 72 hours
- Studies where the administration was started during the surgery; after the surgery
- Surgery performed in the operating room under general anesthesia; surgery in the neonatal ward for minor surgery such as patent ductus arteriosus ligation, surgery for retinopathy of prematurity, positioning of surgical drainage for air leak, thoracocentesis or peritoneal dialysis for acute kidney failure
- Within studies that accepted the use of co‐interventions: studies where investigators allowed co‐interventions for pain management; and studies that obligated its use, as well as by the type of co‐interventions (corticosteroids or nonsteroidal anti‐inflammatory drugs)
- According to drug dose regimen: continuous drug administration; 'as needed' based on signs of pain, discomfort, stress or following medical advisory

We restricted these analyses to the primary outcomes.

#### Sensitivity analysis

Where we identified substantial heterogeneity, we conducted sensitivity analysis to determine if the findings were affected by inclusion of only those trials considered to have used adequate methodology with a low risk of bias (selection and performance bias) by removing the outlying studies. We reported the results of sensitivity analyses for primary outcomes only.

We explored statistical heterogeneity in the outcomes by visually inspecting the forest plots and by removing the outlying studies in a sensitivity analysis (Higgins 2020).

#### Summary of findings and assessment of the certainty of the evidence

We used the GRADE approach, as outlined in the GRADE Handbook (Schünemann 2013), to assess the certainty of evidence for the following (clinically relevant) outcomes.

- Pain assessed with validated methods during the administration of selected drugs
- All‐cause mortality during initial hospitalization
- Major neurodevelopmental disability in children aged 18 to 24 months: cerebral palsy, developmental delay (assessment greater than two standard deviations (SDs) below the mean), intellectual impairment (intelligence quotient (IQ) greater than two SDs below the mean), blindness (vision less than 6/60 in both eyes), or sensorineural deafness requiring amplification (Jacobs 2013)
- Major neurodevelopmental disability (see above) in children three to five years old
- Cognitive and educational outcomes in children more than five years old
- Severe (defined as stage 3 or greater) retinopathy of prematurity in infants examined
- Severe (grade 3 or greater) intraventricular hemorrhage (IVH) on cranial ultrasound

Two review authors (MK, MB) independently assessed the certainty of the evidence for each of the outcomes above. We considered evidence from RCTs as high certainty, downgrading the evidence one level for serious (or two levels for very serious) limitations based upon the following: design (risk of bias), consistency across studies, directness of the evidence, precision of estimates, and presence of publication bias. We used the GRADEpro GDT Guideline Development Tool to create a Summary of findings table to report the certainty of the evidence.

The GRADE approach results in an assessment of the certainty of a body of evidence in one of the following four grades.

- High: we are very confident that the true effect lies close to that of the estimate of the effect;
- Moderate: we are moderately confident in the effect estimate: the true effect is likely to be close to the estimate of the effect, but there is a possibility that it is substantially different;
- Low: our confidence in the effect estimate is limited: the true effect may be substantially different from the estimate of the effect;
- Very low: we have very little confidence in the effect estimate: the true effect is likely to be substantially different from the estimate of effect.

## Results

### Description of studies

See Characteristics of included studies; Characteristics of excluded studies; Characteristics of studies awaiting classification; and Characteristics of ongoing studies.

#### Results of the search

The literature search run in June 2022 yielded a total of 2526 references (2457 after de‐duplication). These references were analyzed using Cochrane's Screen4Me (S4M) platform. S4M categorized 941 references as non‐RCTs (Figure 1). The titles and abstracts of the remaining 1516 were screened by the authors and 1491 were excluded. We evaluated 25 full texts, excluded 17 with reasons (Abiramalatha 2019; Aguirre Corcoles 2003; Anand 1987a; Anand 1987b; Anand 1992; Chiaretti 1997; Chiaretti 2000; ChiCTR‐IPR‐15006112; Dake 1997; Gruber 2001; Karl 2012; Kururattapun 1986; McEwan 2000; Michel 1995; NCT01094522; Pan 2021; Waterworth 1974), and included seven studies our review (Bouwmeester 2001; Bouwmeester 2003a; Bouwmeester 2003b; Czarnecki 2020; Lynn 2000; Van Dijk 2002; Vaughn 1996) (see Figure 2). One study from a trial registry system was classified as 'ongoing' (NCT00004696).

**1:**
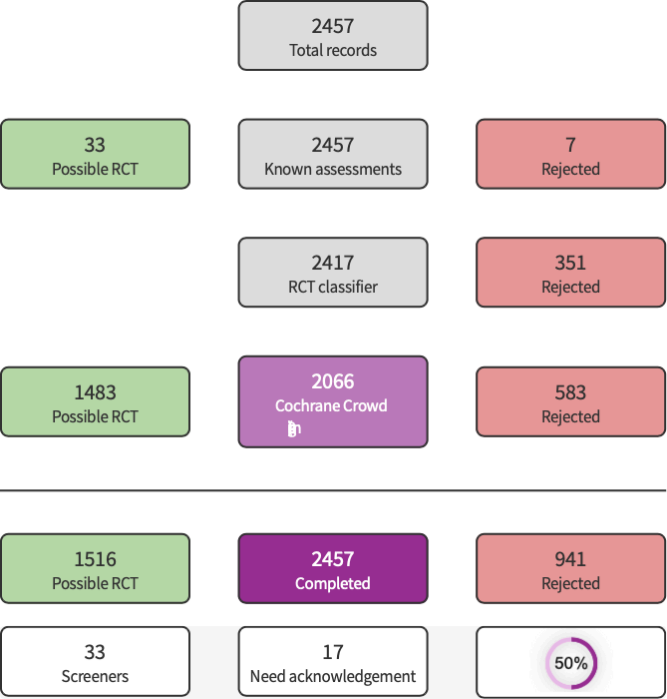
Screen4Me Summary Diagram

**2:**
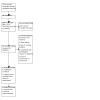
Prisma flow chart

#### Included studies

We included seven studies in the review (Bouwmeester 2001; Bouwmeester 2003a; Bouwmeester 2003b; Czarnecki 2020; Lynn 2000; Van Dijk 2002; Vaughn 1996). See Table 3.

**1:** Table 1 ‐ Overview of included studies

**Study ID**	**Country**	**Sample Size**	**GA (weeks)**	**Intervention**	**Comparison**
**Comparison 1 ‐ Different doses of the same opioid**					
None					
**Comparison 2 ‐ Different routes of administration of the same opioid**					
None					
**Comparison 3 ‐ Continuous infusion versus bolus administration**					
Bouwmeester 2001	The Netherlands	68	Not described	Continuous morphine	Intermittent morphine
Bouwmeester 2003a	The Netherlands	63	38^1^	Continuous morphine	Intermittent morphine
Bouwmeester 2003b	The Netherlands	68	36.1 to 42.1	Continuous morphine	Intermittent morphine
Lynn 2000	United States of America	83	Not described	Continuous morphine	Intermittent morphine
Van Dijk 2002	The Netherlands	181	Not described	Continuous morphine	Intermittent morphine
Vaughn 1996	United States of America	16 and 20^2^	39.8 to 42.4^3^	Continuous fentanyl	Intermittent fentanyl
**Comparison 4 ‐ 'As needed' administration versus 'as scheduled' administration**					
Czarnecki 2020	United States of America	25	36.9 to 39.5	Parent/nurse‐controlled analgesia (morphine)	Continuous infusion (morphine)


GA stands for "Gestational Age"; ID stands for "Identification". We extracted the GA for the said 'Group 1' showed in the study, which included newborn infants. The study had two clearly separate phases: In phase 1, 16 newborn infants were enrolled following randomization; in phase 2, 20 newborn infants were enrolled, without randomization, and therefore their outcome data were not included in this review. Inserted data reported the range of GA for the study for both groups, in both study's phases.

The seven clinical trials included in this review reported data from 504 infants from different settings and primary conditions. In addition, there were differences in the methods, participants, and interventions.

Overall, enrolled patients were initially admitted to neonatal intensive care units after undergoing non‐cardiac, thoracic, or abdominal surgery, which involved the postoperative pain management protocols of each hospital. As far as exclusion criteria among included studies were concerned, most studies considered patients ineligible for inclusion if they had received significant opioid treatment less than six hours before the surgery, received neuromuscular blockade, or suffered from hepatic, renal, neurological, or metabolic pathologies. In addition, one study excluded patients who received mechanical ventilation prior to surgery (Vaughn 1996).

With regard to baseline characteristics among the included studies, four studies included infants up to four weeks of age (Bouwmeester 2001; Bouwmeester 2003a; Bouwmeester 2003b; Van Dijk 2002), and two of these studies only included infants of at least 35 weeks' gestation (Bouwmeester 2003b; Van Dijk 2002). One study included only term infants up to 365 days of age (Lynn 2000). One study included infants between 34 weeks postmenstrual age (PMA) and corrected age of less than 44 weeks (Czarnecki 2020). One study included infants between 36 and 52 weeks PMA (Vaughn 1996).

Morphine and fentanyl were used in six and one trials, respectively. Four studies compared continuous morphine infusion with intermittent morphine boluses every three hours (Bouwmeester 2001; Bouwmeester 2003a; Bouwmeester 2003b; Van Dijk 2002). One study compared continuous morphine infusion with intermittent morphine boluses every one to two hours as needed (Lynn 2000). One study compared continuous morphine infusion (COI) with parent‐ or nurse‐controlled analgesia (PNCA) boluses of morphine (Czarnecki 2020). One study compared continuous fentanyl infusion with fentanyl boluses every two hours (Vaughn 1996).

Pain assessment during postoperative administration of selected drugs were done using validated methods in two of the included studies. In Bouwmeester 2001, two alternative methods were utilized for assessing pain in infants (visual analogue scale and COMFORT scale). Czarnecki 2020 also evaluated the infants' pain, but by using a revised version of the FLACC approach.

We identified only one record in the trial registry platform, which aimed to compare non‐mechanically ventilated infants who received morphine postoperatively as intermittent intravenous bolus doses to those that received continuous intravenous infusion targeted to reach a steady‐state concentration, and to assess effectiveness of analgesia between the two treatment groups of infants (NCT00004696).

#### Excluded studies

The 17 excluded studies following full‐text screening are listed in the Characteristics of excluded studies table. We excluded two studies because of the characteristics of the study design (Anand 1987a; NCT01094522). We excluded nine studies because of the age of the patient population (Aguirre Corcoles 2003; Chiaretti 1997; Chiaretti 2000; Karl 2012; Kururattapun 1986; McEwan 2000; Michel 1995; Pan 2021; Waterworth 1974). We excluded three studies because anesthesia was investigated instead of analgesia (Abiramalatha 2019; ChiCTR‐IPR‐15006112; Gruber 2001). We excluded three studies because of the type of intervention or comparator (Anand 1987a; Anand 1992; Dake 1997).

### Risk of bias in included studies

The overall risk of bias assessment for each study, including all domain evaluations and justifications for judgment, is displayed in the risk of bias section (Characteristics of included studies), on the right side of all forest plots and Figure 3; Figure 4.

**3:**
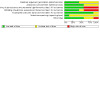
Risk of bias summary

**4:**
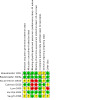
Risk of bias graph

#### Allocation

Four studies did not specify the random sequence generation (Bouwmeester 2003b; Lynn 2000; Van Dijk 2002; Vaughn 1996). Three of these studies did not specify the allocation concealment (Bouwmeester 2003b; Lynn 2000; Vaughn 1996). The remaining three studies had low risk of selection bias (Bouwmeester 2001; Bouwmeester 2003a; Czarnecki 2020).

#### Blinding

Three studies were blinded for both those administering opioids and those assessing the outcomes (Bouwmeester 2001; Bouwmeester 2003a; Van Dijk 2002). In one study, blinding was not specified for the outcome assessors (Vaughn 1996), therefore, it was classified with an unclear risk of bias related to blinding for outcome assessors. The remaining three studies had high risk of performance bias and detection bias (Bouwmeester 2003b; Czarnecki 2020; Lynn 2000).

#### Incomplete outcome data

In Bouwmeester 2001, numbers in the figures did not match the size of each experimental group; moreover, reasons were not clearly stated. The remaining six studies had low risk of attrition bias (Bouwmeester 2003a; Bouwmeester 2003b; Czarnecki 2020; Lynn 2000; Van Dijk 2002; Vaughn 1996).

#### Selective reporting

In Czarnecki 2020, no major discrepancy was identified between the protocol and the final manuscript. The remaining six studies had unclear risk of reporting bias as no protocol was available (Bouwmeester 2001; Bouwmeester 2003a; Bouwmeester 2003b; Lynn 2000; Van Dijk 2002; Vaughn 1996).

#### Other potential sources of bias

In Lynn 2000, the percentage of infant pain scores for each infant was compared between groups rather than the absolute number of scores to compensate for different length of postoperative periods for different surgeries and different absolute numbers of scores based on bolus dosage (this study was assessed as being at high risk of bias). In Vaughn 1996, the study design did not incorporate a systematic approach to wean ventilator support, and therefore interpretation of this observation is difficult (graded as being at unclear risk of bias). Czarnecki 2020 was terminated earlier than planned (graded as being at unclear risk of bias). The remaining four studies had low risk of other potential sources of bias (Bouwmeester 2001; Bouwmeester 2003a; Bouwmeester 2003b; Van Dijk 2002).

### Effects of interventions

See: Table 1; Table 2

#### Comparison 1: Different doses of the same opioid

None of the studies were included in this comparison.

#### Comparison 2: Different routes of administration of the same opioid

None of the studies were included in this comparison.

#### Comparison 3: Continuous infusion versus bolus administration

Six studies were included in this comparison (Bouwmeester 2001; Bouwmeester 2003a; Bouwmeester 2003b; Lynn 2000; Van Dijk 2002; Vaughn 1996); five studies compared continuous morphine infusion with intermittent morphine boluses (Bouwmeester 2001; Bouwmeester 2003a; Bouwmeester 2003b; Lynn 2000; Van Dijk 2002), whereas one study compared continuous fentanyl infusion with fentanyl boluses every two hours (Vaughn 1996). Four studies reported at least one of the outcomes specified in the protocol of this review. See Table 1.

##### Primary outcomes

###### VAS ‐ Pain assessed with validated methods during the administration of selected drugs

Two studies (Bouwmeester 2001; Van Dijk 2002) reported this outcome. We are uncertain whether continuous infusion of opioids reduces pain assessed with visual analogue scale (VAS) compared with bolus administration (MD 0.00, 95% CI ‐0.23 to 0.23; 133 participants, 2 studies; I² = 0; very low‐certainty evidence; Analysis 1.1). Outcome data for Van Dijk 2002 were obtained following contact with study authors.

###### COMFORT ‐ Pain assessed with validated methods during the administration of selected drugs

Two studies (Bouwmeester 2001; Van Dijk 2002) reported this outcome. We are uncertain whether continuous infusion of opioids reduces pain assessed with COMFORT compared with bolus administration (MD ‐0.07, 95% CI ‐0.89 to 0.75; 133 participants, 2 studies; I² = 0; very low‐certainty evidence; Analysis 1.2). Outcome data for Van Dijk 2002 were obtained following contact with study authors.

###### All‐cause mortality during initial hospitalization

None of the included studies reported this outcome.

###### Major neurodevelopmental disability

None of the included studies reported this outcome.

###### Cognitive and educational outcomes in children older than five years old

None of the included studies reported this outcome.

##### Secondary outcomes

###### Hypotension requiring medical therapy

One study (Bouwmeester 2003b) reported no events for this outcome. We are uncertain whether continuous infusion of opioids reduces hypotension requiring medical therapy compared with bolus administration (very low‐certainty evidence; Analysis 1.3).

###### Mechanical ventilation longer than 24 hours

Three studies (Bouwmeester 2001; Bouwmeester 2003a; Vaughn 1996) reported this outcome. Continuous infusion of opioids may reduce mechanical ventilation longer than 24 hours compared with bolus administration (RR 1.62, 95% CI 1.19 to 2.21, RD 0.23, 95% CI 0.09 to 0.38, 147 participants, 3 studies; I² for RR and RD = 0%; low‐certainty evidence; Analysis 1.4, Figure 5). For Bouwmeester 2001, data were reported for mechanical ventilation longer than 36 hours.

**5:**
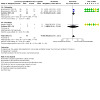
Forest plot for number of infants with mechanical ventilation longer than 24 hours

####### Subgroup analysis 1.4.1 ‐ morphine

Two studies (Bouwmeester 2001; Bouwmeester 2003a) reported this outcome. Continuous infusion of morphine may reduce mechanical ventilation longer than 24 hours compared with bolus administration (RR 1.63, 95% CI 1.20 to 2.21, RD 0.28, 95% CI 0.12 to 0.44, 131 participants, 2 studies; I² for RR and RD = 0%; low‐certainty evidence; Analysis 1.4, Figure 5). For Bouwmeester 2001, data were reported for mechanical ventilation longer than 36 hours.

####### Subgroup analysis 1.4.2 ‐ fentanyl

One study (Vaughn 1996) reported this outcome. We are uncertain whether continuous infusion of fentanyl reduces mechanical ventilation longer than 24 hours compared with bolus administration (RR 1.29, 95% CI 0.10 to 17.14, RD 0.03, 95% CI ‐0.30 to 0.36, 16 participants, 1 study; I² not applicable; very low‐certainty evidence; Analysis 1.4, Figure 5).

Within Comparison 3, no studies reported: all‐cause neonatal mortality; episodes of bradycardia; retinopathy of prematurity; intraventricular hemorrhage; periventricular leukomalacia; necrotizing enterocolitis; bronchopulmonary dysplasia/chronic lung disease; constipation; focal gastrointestinal perforation; duration of mechanical ventilation; duration of oxygen supplementation; hospital stay; time to full enteral feeding; cost of neonatal care.

#### Comparison 4: 'as needed' administration (e.g. based on pain scales) versus 'as scheduled' administration (e.g. a predefined time interval)

One study (Czarnecki 2020) was included in the comparison, however, it reported none of the outcomes specified in the protocol of this review (see Table 2).

## Discussion

### Summary of main results

In this review, we included seven studies with a total of 504 newborn infants. We identified no studies comparing different doses of the same opioid, or different routes. Six studies compared continuous opioid infusion with intermittent opioid boluses, either using morphine (five studies) or fentanyl (one study); one study compared continuous morphine infusion with parent‐ or nurse‐controlled analgesia boluses of morphine, however, reported none of the outcomes of this review.

Evidence from two studies in 133 infants is uncertain whether continuous opioid infusion reduces pain compared with intermittent opioid boluses. Neither did the included studies report on the other primary outcomes of this review, i.e. all‐cause mortality during initial hospitalization, major neurodevelopmental disability, or cognitive and educational outcomes in children older than five years old. Evidence from one study in 62 infants, with no corresponding events, is uncertain whether continuous opioid infusion reduces hypotension requiring medical therapy compared with intermittent opioid boluses. None of the remaining outcomes were reported in any of the trials.

### Overall completeness and applicability of evidence

A total of 504 newborns have been enrolled into seven clinical trials to compare different systemic opioid regimens, mainly continuous infusion and intermittent boluses of morphine. Study authors often assessed infant pain but using different scales, and they rarely reported other important outcomes such as long‐term neurodevelopment. We identified one possibly ongoing study, which was categorized as 'awaiting classification' due to the uncertainty regarding its trial status. More trials comparing the same systemic regimens and assessing critical outcomes are necessary for reaching meaningful conclusions about postoperative pain management in newborns.

### Quality of the evidence

Following the GRADE approach, the overall certainty of evidence for the reported outcomes for postoperative systemic opioid administration is very low to low (See Table 1). The few reported outcomes were all downgraded (one level) for limitations in study design owing to the unclear risk of attrition or reporting bias. The outcome assessing the number of infants with mechanical ventilation longer than 24 hours was further downgraded (one level) for imprecision owing to the small sample size of one included study, and thus was rated as having low certainty. The other outcomes (pain assessment by different scales) were further downgraded (two levels) for imprecision because only one study was included in each analysis, and thus were rated as having very low certainty. We did not use funnel plots to evaluate publication bias because there were fewer than 10 studies that met the inclusion criteria of this Cochrane Review.

### Potential biases in the review process

Throughout the review process, we adhered to the protocols and procedures endorsed by Cochrane and the MECIR standards to alleviate any potential procedural bias. Moreover, there were no deviations from the original protocol.

The reporting of the outcomes significantly varied among the included clinical trials, and we did not anticipate this issue. This led to a limited number of analyses of the included studies in terms of quantitative and qualitative evaluation, which evidently do not directly reflect the whole scientific literature. This is a potential limitation of this review since, for the most part, the reported outcomes did not align with our choice of primary and secondary outcomes. For instance, most studies assessed the association between morphine, fentanyl, or other opioid administration regimens and hormonal and metabolic stress response (including the dosage of plasma concentrations of norepinephrine, epinephrine, and their metabolites). We were successful in obtaining additional outcome data from study authors for one study (Van Dijk 2002).

### Agreements and disagreements with other studies or reviews

There are few randomized trials or other studies evaluating the effectiveness and safety of the systemic opioids for postoperative pain in neonates. The lack of studies evaluating pain management in newborn infants may be associated with the inherent difficulties in assessing pain in a population that typically cannot verbalize their feelings and needs. However, prior to our review, few non‐Cochrane systematic reviews have summarized the available literature on pain management in neonates. At the moment, there is another Cochrane review (Kinoshita 2021) being worked on that is aiming to similarly evaluate the effectiveness and safety of opioids in managing postoperative pain in neonates, but comparing opioids to any other analgesics.

The most complete systematic review with a series of meta‐analyses included 22 randomized clinical trials assessing the effectiveness and side effect profile of tramadol for postoperative pain relief in children and adolescents undergoing different surgical procedures (Schnabel 2015). It turned out that the evidence regarding the use of tramadol for postoperative pain in children is low or very low essentially because of small samples sizes and methodological drawbacks. In addition, the evaluation of adverse events associated with tramadol was not possible due to the lack of reporting of this outcome. However, the applicability of these findings to neonates is likely to be limited.

Another review, which only included randomized, double‐blind clinical trials comparing treatment with morphine with a placebo or active control intervention for efficacy on postoperative pain in pediatrics, only found significant improvements in the analgesic efficacy‐related outcomes when morphine was compared with inactive control interventions (Duedahl 2007). Moreover, the study did not identify any dose‐response effect among the included studies. According to the review, which did not focus on newborns, the most frequently observed morphine‐related adverse events were vomiting and sedation.

## Authors' conclusions

**Implications for practice**

Limited evidence is available on continuous infusion compared to intermittent boluses of systemic opioids. We are uncertain whether continuous opioid infusion reduces pain compared with intermittent opioid boluses; none of the studies reported the other primary outcomes of this review, i.e. all‐cause mortality during initial hospitalization, major neurodevelopmental disability, or cognitive and educational outcomes in children older than five years old. Only one small study reported on morphine infusion with parent‐ or nurse‐controlled analgesia.

**Implications for research**

Recently completed and future trials should report robust and long‐term outcomes in infants exposed to different systemic dosing regimens, in both term and preterm newborn infants. Blinding should be performed and protocols published in advance. Observational studies might provide useful information regarding potential harms.

## What's new

**Table:** 

**Date**	**Event**	**Description**
6 April 2023	New citation required but conclusions have not changed	Republished with different license type.
6 April 2023	Amended	Republished with different license type.

## History

Protocol first published: Issue 5, 2021 Review first published: Issue 1, 2023

## Appendices

### Appendix 1. Search strategies

#### Pubmed

#1 (((infant, newborn[MeSH] OR newborn*[TIAB] OR "new born"[TIAB] OR "new borns"[TIAB] OR "newly born"[TIAB] OR baby*[TIAB] OR babies*[TIAB] OR premature[TIAB] OR prematurity[TIAB] OR preterm[TIAB] OR "pre term"[TIAB] OR “low birth weight”[TIAB] OR "low birthweight"[TIAB] OR VLBW[TIAB] OR LBW[TIAB] OR infan*[TIAB] OR neonat*[TIAB])))

#2 (((((morphine OR diamorphine OR fentanyl OR alfentanil OR sufentanil OR pethidine OR meperidine OR codeine OR methadone))) OR ("Narcotics"[Majr] OR "Analgesia"[Majr] OR sedation[Title/Abstract] OR opioid*[Title/Abstract] OR remifentanil)) OR (((((((("Morphine"[Mesh]) OR "Heroin"[Mesh]) OR "Fentanyl"[Mesh]) OR "Alfentanil"[Mesh]) OR "Sufentanil"[Mesh]) OR "Meperidine"[Mesh]) OR "Codeine"[Mesh]) OR "Methadone"[Mesh] OR “Remifentanil”[Mesh]))

#3 ("Surgical Procedures, Operative"[Mesh] OR surgery[TIAB] OR surgical[TIAB] OR "postoperat*"[TIAB] OR "post operat*"[TIAB] OR "postsurg*"[TIAB] OR "post surg*"[TIAB] OR operative[TIAB] OR operation*[TIAB] OR ligation*[TIAB] OR repair[TIAB])

#4 ((((randomized controlled trial [pt] OR controlled clinical trial [pt] OR randomized [tiab] OR placebo [tiab] OR drug therapy [sh] OR randomly [tiab] OR trial [tiab] OR groups [tiab])) NOT (animals[MH] NOT humans[MH])))

#5 #1 AND #2 AND #3 AND #4

#### Cochrane Library/CENTRAL via Wiley

#1 MeSH descriptor: [Infant, Newborn] explode all trees

#2 (infan* or newborn* or "new born" or "new borns" or "newly born" or neonat* or baby* or babies or premature or prematures or prematurity or preterm* or "pre term" or premies or "low birth weight" or "low birthweight" or VLBW or LBW or ELBW or NICU):ti,ab,kw (Word variations have been searched)

#3 (morphine OR diamorphine OR fentanyl OR alfentanil OR sufentanil OR pethidine OR meperidine OR codeine OR methadone OR remifentanil):ti,ab,kw (Word variations have been searched)

#4 (surgery OR surgical OR postoperat* OR "post operat*" OR postsurg* OR "post surg*" OR operative OR operation*):ti,ab,kw (Word variations have been searched)

#5 MeSH descriptor: [Surgical Procedures, Operative] explode all trees

#6 #1 OR #2

#7 #4 OR #5

#8 #3 AND #6 AND #7

#### CINAHL via EBSCOHost

#1 (infant or infants or infant’s or infantile or infancy or newborn* or "new born" or "new borns" or "newly born" or neonat* or baby* or babies or premature or prematures or prematurity or preterm or preterms or "pre term" or premies or "low birth weight" or "low birthweight" or VLBW or LBW)

#2 (morphine OR diamorphine OR fentanyl OR alfentanil OR sufentanil OR pethidine OR meperidine OR codeine OR methadone OR MH morphine OR MH diamorphine OR MH fentanyl OR MH alfentanil OR MH sufentanil OR MH pethidine OR MH meperidine OR MH codeine OR MH methadone OR MH remifentanil OR MJ narcotics OR MJ sedation OR MJ analgesia OR TI opioid* OR AB opioid*)

#3 (MH "Surgery, Operative+")

#4 surgery OR surgical OR postoperat* OR "post operat*" OR postsurg* OR "post surg*" OR operative OR operation*

#5 #3 OR #4

#6 (randomized controlled trial OR controlled clinical trial OR randomized OR randomised OR placebo OR clinical trials as topic OR randomly OR trial OR PT clinical trial)

#7 #1 AND #2 AND #5 AND #6

### Appendix 2. Risk of bias tool

We used the standard methods of Cochrane and Cochrane Neonatal to assess the methodological quality of the trials. For each trial, we sought information regarding the method of randomization, blinding, and reporting of all outcomes of all the infants enrolled in the trial. We assessed each criterion as being at a low, high, or unclear risk of bias. Two review authors separately assessed each study. We resolved any disagreements by discussion. We added this information to the 'Characteristics of included studies' table. We evaluated the following issues and entered the findings into the Risk of bias table.

#### 1. Sequence generation (checking for possible selection bias). Was the allocation sequence adequately generated?

For each included study, we will categorize the method used to generate the allocation sequence as:

1. low risk (any truly random process, e.g. random number table; computer random number generator);
2. high risk (any non‐random process, e.g. odd or even date of birth; hospital or clinic record number); or
3. unclear risk.

#### 2. Allocation concealment (checking for possible selection bias). Was allocation adequately concealed?

For each included study, we will categorize the method used to conceal the allocation sequence as:

1. low risk (e.g. telephone or central randomization; consecutively numbered, sealed, opaque envelopes);
2. high risk (open random allocation; unsealed or non‐opaque envelopes, alternation; date of birth); or
3. unclear risk

#### 3. Blinding of participants and personnel (checking for possible performance bias). Was knowledge of the allocated intervention adequately prevented during the study?

For each included study, we will categorize the methods used to blind study participants and personnel from knowledge of which intervention a participant received. We will assess blinding separately for different outcomes or class of outcomes. We will categorize the methods as:

- low risk, high risk, or unclear risk for participants; and
- low risk, high risk, or unclear risk for personnel.

#### 4. Blinding of outcome assessment (checking for possible detection bias). Was knowledge of the allocated intervention adequately prevented at the time of outcome assessment?

For each included study, we will categorize the methods used to blind outcome assessment. We will assess blinding separately for different outcomes or class of outcomes. We will categorize the methods as:

1. low risk for outcome assessors;
2. high risk for outcome assessors; or
3. unclear risk for outcome assessors.

#### 5. Incomplete outcome data (checking for possible attrition bias through withdrawals, dropouts, protocol deviations). Were incomplete outcome data adequately addressed?

For each included study and for each outcome, we will describe the completeness of data including attrition and exclusions from the analysis. We will note whether attrition and exclusions were reported, the numbers included in the analysis at each stage (compared with the total randomized participants), reasons for attrition or exclusion where reported, and whether missing data were balanced across groups or were related to outcomes. Where sufficient information is reported or supplied by the trial authors, we will re‐include missing data in the analyses. We will categorize the methods as:

1. low risk (< 20% missing data);
2. high risk (≥ 20% missing data); or
3. unclear risk.

#### 6. Selective reporting bias. Are reports of the study free of the suggestion of selective outcome reporting?

For each included study, we will describe how we investigated the possibility of selective outcome reporting bias and what we found. For studies in which study protocols were published in advance, we will compare prespecified outcomes versus outcomes eventually reported in the published results. If the study protocol was not published in advance, we will contact study authors to gain access to the study protocol. We will assess the methods as:

1. low risk (where it is clear that all of the study's prespecified outcomes and all expected outcomes of interest to the review have been reported);
2. high risk (where not all the study's prespecified outcomes have been reported; one or more reported primary outcomes were not prespecified outcomes of interest and are reported incompletely and so cannot be used; the study fails to include results of a key outcome that would have been expected to have been reported); or
3. unclear risk.

#### 7. Other sources of bias. Was the study apparently free of other problems that could put it at high risk of bias?

For each included study, we will describe any important concerns we had about other possible sources of bias (e.g. whether there was a potential source of bias related to the specific study design or whether the trial was stopped early due to some data‐dependent process). We will assess whether each study was free of other problems that could put it at risk of bias as:

1. low risk;
2. high risk;
3. unclear risk.

If needed, we plan to explore the impact of the level of bias by undertaking sensitivity analyses.

## Data and analyses

**Comparison 1:** Continuous infusion versus bolus administration

Outcome or subgroup title	No. of studies	No. of participants	Statistical method	Effect size
1.1 Pain assessed with visual analogue scale (VAS) during the administration of selected drugs (neonates from 0 to 4 weeks)	2	133	Mean Difference (IV, Fixed, 95% CI)	0.00 [‐0.23, 0.23]
1.2 Pain assessed with COMFORT scale during the administration of selected drugs (neonates from 0 to 4 weeks)	2	133	Mean Difference (IV, Fixed, 95% CI)	‐0.07 [‐0.89, 0.75]
1.3 Hypotension requiring medical therapy	1		Risk Difference (IV, Fixed, 95% CI)	Totals not selected
1.4 Mechanical ventilation longer than 24 hours	3	147	Risk Ratio (IV, Fixed, 95% CI)	1.62 [1.19, 2.21]
1.4.1 Morphine	2	131	Risk Ratio (IV, Fixed, 95% CI)	1.63 [1.20, 2.22]
1.4.2 Fentanyl	1	16	Risk Ratio (IV, Fixed, 95% CI)	1.29 [0.10, 17.14]

## Characteristics of studies

### Characteristics of included studies [ordered by study ID]

**Table:** Bouwmeester 2001

***Study characteristics***		
Methods	**Study design:** Double‐blind, randomized clinical trial **Study grouping:** Continuous morphine versus three‐hourly placebo or intermittent morphine with placebo infusion	
Participants	**Baseline Characteristics** **Inclusion criteria:** Children aged 0 to 3 years, admitted to the pediatric surgical intensive care unit after non‐cardiac thoracic and abdominal surgery **Exclusion criteria:** Author excluded patients if they had received analgesic or sedative drugs less than 6 hours before surgery, if they were receiving neuromuscular blockade or if they suffered from hepatic, renal or neurological disorders or altered muscle tone. **Pretreatment:** Anesthesia was induced with thiopentone or by inhalation of halothane in oxygen. Fentanyl 5 mcg/kg was given before orotracheal intubation, which was facilitated with atracurium 0.5‐1 mcg/kg or suxamethonium 2 mcg/kg.	
Interventions	**Intervention Characteristics** **Continuous infusion versus bolus administration** ***Dose regimen*:** Two groups (continuous morphine versus intermittent morphine) ***Description of procedure*:** One group received continuous morphine (10 mcg/kg/h) while the other group received a continuous placebo infusion combined with three‐hourly intravenous doses of 30 mcg/kg. All participants received an intravenous loading dose of morphine (100 mcg/kg).	
Outcomes	*Pain assessed with validated methods during the administration of selected drugs* **Outcome type**: Continuous outcome Nurses performed regular assessments before surgery (baseline) and every 3 h up to 36 h after surgery. Nursing interventions included pain assessment using a VAS and the COMFORT scale.	
Identification	**Sponsorship source:** The study was supported by the Dutch Research Council and the Sophia Foundation for Medical Research. **Country:** The Netherlands **Setting:** Pediatric Intensive Care Unit **Comments:** None **Authors names:** Nancy J. Bouwmeester, K.J.S. Anand, Monique van Dijk, Wim C. J. Hop, F. Boomsma, and Dick Tibboel **Institution:** Department of Anesthesiology and Pediatric Surgery, Sophia Children's Hospital **Email:** Not provided **Address:** Sophia Children's Hospital, University Hospital Rotterdam, Dr Molewaterplein 60, 3015 GJ Rotterdam, The Netherlands	
Notes		
***Risk of bias***		
**Bias**	**Authors' judgement**	**Support for judgement**
Random sequence generation (selection bias)	Low risk	"The pharmacists prepared all study drugs, and the strata‐specific schedules for randomization". Computer‐generated
Allocation concealment (selection bias)	Low risk	Clinical staff blinded to allocation
Blinding of participants and personnel (performance bias) All outcomes	Low risk	"Pharmacists prepared all study drugs, and the strata‐specific schedules for randomization and the clinical staff were blinded to the study group allocation until data collection was complete." Blinded
Blinding of outcome assessment (detection bias) All outcomes	Low risk	"Clinical staff were blinded to the study group allocation until data collection was complete".
Incomplete outcome data (attrition bias) All outcomes	Unclear risk	"All enrolled patients were included in an intention‐to‐treat analysis. Nine patients dropped out during the study (five in CM, four in IM) because of the loss of arterial access (seven), the need for neuromuscular blockade (one) and one postoperative death 3 h after surgery." N in the figures did not match the size of each experimental group and reasons were not clearly stated.
Selective reporting (reporting bias)	Unclear risk	No registry stated so unable to compare to protocol
Other bias	Low risk	None

**Table:** Bouwmeester 2003a

***Study characteristics***		
Methods	**Study design:** Double‐blind, randomized clinical trial **Study grouping:** Continous morphine versus three‐hourly placebo or intermittent morphine with placebo infusion. Additional morphine was administered on guidance of pain outcomes.	
Participants	**Baseline Characteristics:** Neonates aging from 0 to 4 weeks (gestational age between 35 to 42 weeks; body weight 1500 g or more) **Inclusion criteria:** Included neonates were admitted to the pediatric surgical intensive care unit following non‐cardiac thoracic and abdominal surgery. **Exclusion criteria:** Patients were excluded if they had received morphine < 6 h before surgery, or suffered from hepatic, renal or neurological disorders. **Pretreatment:** Anesthesia was induced intravenously with 3 to 5 mg/kg thiopentone or by inhalation with halothane in oxygen. Fentanyl at 5 mcg/kg was given before orotracheal intubation, which was facilitated with 0.5 to 1 mg/kg atracurium or 2 mg/kg suxamethonium.	
Interventions	**Intervention Characteristics** ***Dose regimen*:** Continous morphine infusion (10 mcg/kg/h) versus intermittent morphine (10 mcg/kg every 3 hours) ***Description of procedure*:** All neonates received a dose of morphine hydrochloride 100 mcg/kg at the end of surgery and were randomly allocated to equivalent intravenous doses of continuous morphine infusions (10 mcg/kg/hour) or intermittent morphine boluses (10 mcg/kg/3 hours).	
Outcomes	*Pain assessed with validated methods during the administration of selected drugs* **Outcome type**: Continuous outcome Pain was assessed by nurses trained in the use of the behavioral part of the COMFORT scale (CS), the total score of which can range from 6 to 30, and a 0 ± 10 visual analogue scale (VAS).	
Identification	**Sponsorship source:** The study was supported by the Netherlands Research Council and the Sophia Foundation for Medical Research. **Country:** The Netherlands **Setting:** Pediatric Intensive Care Unit **Comments:** None **Authors names:** Nancy J. Bouwmeester, Wim C. J. Hop, Monique van Dijk, K.J.S. Anand, John N. van den Anker, and Dick Tibboel **Institution:** Department of Anesthesiology and Pediatric Surgery, Sophia Children's Hospital **Email:** j.bouwmeester.1@erasmusmc.nl **Address:** Sophia Children's Hospital, University Hospital Rotterdam, Dr Molewaterplein 60, 3015 GJ Rotterdam, The Netherlands	
Notes		
***Risk of bias***		
**Bias**	**Authors' judgement**	**Support for judgement**
Random sequence generation (selection bias)	Low risk	"They were randomly assigned to receive either i.v. continuous morphine (CM) or intermittent morphine (IM)." Computer‐generated (stratification)
Allocation concealment (selection bias)	Low risk	"The pharmacist prepared all study drugs and strata‐specific schedules for randomization." "For each age group, the boxes containing the study drugs were numbered consecutively and used in sequence. Each patient received a study number consisting of a number of the age group (I ± IV) and a sequence number (1 ± 68)." Concealed
Blinding of participants and personnel (performance bias) All outcomes	Low risk	"For children in the CM group this was followed by a morphine infusion 10 mg kg^‐1^ h^‐1^, combined with 3‐hourly i.v. placebo (saline) boluses." "Pharmacist prepared all study drugs and strata‐specific schedules for randomization." "The amount of glucose and the volume of fluid was the same in both treatment groups." Blinded
Blinding of outcome assessment (detection bias) All outcomes	Low risk	"The clinical staff were blinded to the study group allocation until data collection was complete."
Incomplete outcome data (attrition bias) All outcomes	Low risk	"Logistic and laboratory problems resulted in missing data for several of the 197 included patients." "All 204 patients were included in an intention‐to‐treat analysis." Limited missing data
Selective reporting (reporting bias)	Unclear risk	No registry reported so unable to compare to protocol
Other bias	Low risk	None

**Table:** Bouwmeester 2003b

***Study characteristics***		
Methods	**Study design:** Double‐blind, randomized clinical trial **Study grouping:** Continuous morphine versus three‐hourly placebo or intermittent morphine with placebo infusion. Additional morphine was administered on the guidance of clinicians if they considered the infant was in pain.	
Participants	**Baseline Characteristics:** Neonates aging from 0 to 4 weeks (gestational age between 35 and 42 weeks; body weight 1500 g or more) Overall **Inclusion criteria:** Included neonates were admitted to the surgical intensive care unit following thoracic or abdominal surgery. **Exclusion criteria:** Neonates with neurological, renal, or hepatic dysfunction, or with opioid therapy less than 6 hours prior to surgery were excluded. **Pretreatment:** Anesthesia was induced intravenously with 3 to 5 mg/kg thiopentone or by inhalation with halothane in oxygen. Fentanyl at 5 mcg/kg was given before orotracheal intubation, which was facilitated with 0.5 to 1 mg/kg atracurium or 2 mg/kg suxamethonium.	
Interventions	**Intervention Characteristics** Continuous infusion versus bolus administration ***Dose regimen*:** Intravenous doses of continuous morphine infusions (10 mcg/kg/hour) or intermittent morphine boluses (0 mcg/kg/3 hours) ***Description of procedure*:** All neonates received a dose of morphine hydrochloride 100 mcg/kg at the end of surgery and were randomly allocated to equivalent intravenous doses of continuous morphine infusions (10 mcg/kg/hour) or intermittent morphine boluses (0 mcg/kg/3 hours).	
Outcomes	*Pain assessed with validated methods during the administration of selected drugs* **Outcome type**: Continuous outcome Pain was assessed by trained nurses before surgery and every 3 h for 24 h after surgery using the validated behavioral Comfort scale (CS; total scores range from 6 to 30) and visual analogue scale (VAS; ranging from 0 to 10).	
Identification	**Sponsorship source:** The study was supported by the Netherlands Research Council and the Sophia Foundation for Medical Research. **Country:** The Netherlands **Setting:** Pediatric Intensive Care Unit **Comments:** None **Authors names:** Nancy J. Bouwmeester, Wim C. J. Hop, Monique van Dijk, K.J.S. Anand, John N. van den Anker, and Dick Tibboel **Institution:** Department of Anesthesiology and Pediatric Surgery, Sophia Children's Hospital **Email:** j.bouwmeester.1@erasmusmc.nl **Address:** Sophia Children's Hospital, University Hospital Rotterdam, Dr Molewaterplein 60, 3015 GJ Rotterdam, The Netherlands	
Notes		
***Risk of bias***		
**Bias**	**Authors' judgement**	**Support for judgement**
Random sequence generation (selection bias)	Unclear risk	"All neonates received a dose of morphine hydrochloride 100 µg/kg at the end of surgery and were randomly allocated to equivalent intravenous doses of continuous morphine infusions (CM, 10 µg/kg per hour) or intermittent morphine boluses (IM, 30 µg/kg per 3 hours)." Not specified further
Allocation concealment (selection bias)	Unclear risk	Not specified
Blinding of participants and personnel (performance bias) All outcomes	High risk	Not a blinded trial. Not described in the paper; the difference in the protocol can be visually noticed.
Blinding of outcome assessment (detection bias) All outcomes	High risk	"Pain was assessed by trained nurses before surgery and every 3 h for 24 h after surgery using the validated behavioral Comfort scale (CS; total scores range from 6 to 30) and visual analogue scale (VAS; ranging from 0 to 10)." Not a blinded trial. The difference in the protocol is visually apparent; other outcomes besides pain assessment were objective (plasma concentrations, etc.).
Incomplete outcome data (attrition bias) All outcomes	Low risk	"Six patients were excluded from morphine analysis, five because of detectable plasma morphine levels at baseline and one because of loss of the arterial line at the end of surgery. The five patients with detectable morphine concentrations at time 0 had received morphine more than 6 h before surgery." "Hemodynamic data could not be analyzed in 3 of the 68 neonates due to loss of the arterial line during the study." No major concern
Selective reporting (reporting bias)	Unclear risk	No registry reported so unable to compare with protocol
Other bias	Low risk	None

**Table:** Czarnecki 2020

***Study characteristics***		
Methods	**Study design:** Randomized clinical trial **Study grouping:** Morphine parent/nurse‐controlled analgesia versus continuous opioid infusion	
Participants	**Baseline Characteristics** **Inclusion criteria:** Infants born no earlier than 34 weeks postmenstrual age and having a corrected age of no greater than 44 weeks at the time of enrollment, had a birth or current weight of at least 2 kg, underwent an abdominal or thoracic surgical procedure conducted by the pediatric general and thoracic surgery team, and expected to require opioids for at least 24 hours after the surgical procedure based on the opinion of the surgeon. Additionally, at least one parent was required to be able to read and speak English. **Exclusion criteria:** Authors excluded patients if they required vasopressors, had significant prior opioid exposure, or were expected to be chemically paralyzed after surgery. **Pretreatment:**	
Interventions	**Intervention Characteristics** **Continuous infusion versus bolus administration** ***Dose regimen*:** Morphine parent/nurse‐controlled analgesia versus continuous opioid infusion (0.030 mg/kg/h) ***Description of procedure*:** A 250 mcg/mL solution of parent/nurse‐controlled morphine, 0.010 to 0.0125 mg/kg/h basal rate (0.060 mg/kg/h total maximum) was ordered; doses were rounded to the nearest microgram. Parents were instructed about signs of pain and told to: push the parent/nurse‐controlled analgesia button only when the child was awake and in pain; to never push the button when the child was asleep; and to notify their nurse if they felt their child’s pain was not well controlled. For the continuous opioid infusion group, morphine was ordered at 0.030 mg/kg/h, with a 0.030 mg/kg/h nurse bolus available each hour to a maximum of 0.060 mg/kg/h total. Parents were instructed on signs of pain and to notify their nurse if their child’s pain was not well controlled.	
Outcomes	*Pain assessed with validated methods during the administration of selected drugs* **Outcome type**: Continuous outcome Average pain intensity over the time of the study. Participant pain intensity was assessed using the Revised‐Face, Legs, Activity, Cry, Consolability (Revised‐FLACC) scale.	
Identification	**Sponsorship source:** The project described was supported by the National Center for Advancing Translational Sciences, National Institutes of Health. **Country:** United States of America **Setting:** Neonatal Intensive Care Unit **Comments:** None **Authors names:** Michelle L. Czarnecki, Keri Hainsworth, Pippa M. Simpson, Marjorie J. Arca, Michael R. Uhing, Liyun Zhang, Ann Grippe, Jaya Varadarajan, Lynn M. Rusy, Mary Firary, StevenJ. Weisman **Institution:** Jane B. Pettit Pain and Headache Center, Children’s Hospital of Wisconsin, Milwaukee, Wisconsin **Email:** mczarnecki@chw.org **Address:** Jane B. Pettit Pain and Headache Center, Children’s Hospital of Wisconsin, P.O. Box 1997, MS 792, Milwaukee, WI 53201	
Notes	*Mari Kinoshita* on 29/08/2021 19:15 **Included** Method matches that of 3496 ‐ NCT 2013 (registry number not clearly stated in paper)	
***Risk of bias***		
**Bias**	**Authors' judgement**	**Support for judgement**
Random sequence generation (selection bias)	Low risk	"The randomization scheme was created by a statistician on the research team using Windows version 6.0 of “rand.exe” (http://block‐stratified‐ randomization.software.informer.com/). The clinical research coordinator used the scheme to assign participants to groups." Computer‐generated
Allocation concealment (selection bias)	Low risk	"The clinical research coordinator used the scheme to assign participants to groups." Concealed
Blinding of participants and personnel (performance bias) All outcomes	High risk	"Based on our current practice, PNCA patients were managed by the APS, and COI patients were managed by the neonatology team. COI patients were checked on". Not blinded
Blinding of outcome assessment (detection bias) All outcomes	High risk	"This study used an unblinded design." Not blinded
Incomplete outcome data (attrition bias) All outcomes	Low risk	"There were 371 screened patients, with the final sample consisting of 25 infants, 16 in the PNCA group and nine in the COI group (CONSORT diagram shown in Fig. 1). Four participants from each group were removed from the study early, but all eight had at least one post‐randomization outcome and were included in the modified intent‐to‐treat analyses." No major concern
Selective reporting (reporting bias)	Low risk	Protocol registered as NCT01823497
Other bias	Unclear risk	"The current trial was originally designed to randomize 30 patients into each study arm based on an a priori power analysis. As stated previously, constraints in funding, and difficulty recruiting, required early termination of the trial. Although most parents were open to the idea when initially approached, they declined enrollment owing to the randomized nature of the study. In fact, 39% (28 of 71 approached) declined enrollment, with many stating they wanted their surgeon to make decisions regarding their infant’s pain management. Another reason for the small sample size was the number of enrolled patients who, despite their infants originally seeming likely to require a PNCA/COI for post‐surgical pain, ultimately had an epidural placed during surgery. Of the 36 who were consented and randomized, 28% (n = 10) received an epidural." Early termination of the study

**Table:** Lynn 2000

***Study characteristics***		
Methods	**Study design:** Randomized clinical trial **Study grouping:** Continuous infusion to a targeted morphine concentration of 20 ng/mL or as intermittent bolus doses, as needed	
Participants	**Baseline Characteristics** **Inclusion criteria:** Infants scheduled for non‐cardiac surgeries involving postoperative hospital admission **Exclusion criteria:** Infants selected were those not expected to need postoperative mechanical ventilation, infants born prematurely (less than 36 weeks gestation), those with abnormal renal or hepatic function, those whose surgery involved tracheobronchial reconstruction or closure of large omphalocele or gastroschisis defects, and those with prenatal or preoperative exposure to opiates. **Pretreatment:** Anesthetic management was not standardized, but included volatile agents and muscle relaxant. Fentanyl (1 to 2 mcg/kg) was used in five cases. Ten infants had single‐dose caudal bupivacaine for intraoperative analgesia.	
Interventions	**Intervention Characteristics** **Continuous infusion versus bolus administration** ***Dose regimen*:** Continuous infusion to a targeted morphine concentration of 20 ng/mL or as intermittent bolus doses, as needed ***Description of procedure*:** Intravenous morphine sulfate, either by intermittent bolus dosage (0.05 mg/kg) ordered every 1 to 2 h as needed or by continuous intravenous infusion targeted to not exceed a steady‐state serum morphine concentration of 20 ng/mL	
Outcomes	*Pain assessed with validated methods during the administration of selected drugs* **Outcome type**: Continuous outcome All infants had serial pain assessments using a modified infant pain rating instrument. Modified infant pain scores were assessed every 4 h and prior to any bolus dose of morphine in both groups. *Duration of oxygen supplementation (days)* **Outcome type**: Continuous outcome Ventilatory effects of morphine in postoperative infants. See table 3.	
Identification	**Sponsorship source:** Supported by Orphan Product Grant from the Food and Drug Administration **Country:** United States of America **Setting:** Neonatal Intensive Care Unit **Comments:** None **Authors names:** Anne M. Lynn, Mary Kay Nespeca, Susan L. Bratton, Danny D. Shen **Institution:** Department of Anesthesiology, University of Washington Schools of Medicine and Pharmacy **Email:** Not provided **Address:** Department of Anesthesiology, University of Washington Schools of Medicine and Pharmacy, Seattle, WA, USA	
Notes		
***Risk of bias***		
**Bias**	**Authors' judgement**	**Support for judgement**
Random sequence generation (selection bias)	Unclear risk	"Infants were randomized". Not specified
Allocation concealment (selection bias)	Unclear risk	"Study enrollment occurred before or during surgery." Not specified
Blinding of participants and personnel (performance bias) All outcomes	High risk	"Placebo infusions were not given to the bolus‐treated group" Not specified
Blinding of outcome assessment (detection bias) All outcomes	High risk	"Placebo infusions were not given to the bolus‐treated group, so bias in pain evaluation by nurses was possible, but use of the modified infant pain scale standardized their assessments and evaluated parameters easily quantified." Not specified
Incomplete outcome data (attrition bias) All outcomes	Low risk	"Seven infants withdrew during the study, three when intravenous catheters infiltrated and oral analgesic medication was adequate, two when parents decided they did not want additional venipunctures, one infant required no morphine or other opiate postoperatively, and one infant was switched to a fentanyl infusion because of severe pruritus during morphine infusion. Except for the last two infants described above, all infant pain scores from the 88 infants up to the time of study termination were included in the analgesia assessment as outlined in the methods. This left 83 infants, 56 in the infusion group and 27 in the intermittent bolus group with morphine serum concentrations and ventilatory data for analysis." No major concern
Selective reporting (reporting bias)	Unclear risk	No registry number reported so unable to compare to protocol
Other bias	High risk	"12 were considered inadequate analgesia. The percentage of infant pain scores > 15, and of scores < 12 for each infant were compared between groups rather than the absolute number of scores to compensate for different length of postoperative periods for different surgeries and different absolute numbers of scores based on bolus dosage. All infants were included in..." See quote

**Table:** Van Dijk 2002

***Study characteristics***		
Methods	**Study design:** Double‐blind, randomized clinical trial **Study grouping:** Intermittent morphine versus continuous morphine	
Participants	**Baseline Characteristics** **Inclusion criteria:** Children aged 0–3 years, admitted for major abdominal or thoracic surgery. In addition, included patients were neonates (greater than or equal to 35 weeks gestation and body weight greater than or equal to 1500 g) and infants aged up to 3 years. **Exclusion criteria:** Exclusion criteria were use of analgesic or sedative co‐medication (e.g. acetaminophen or midazolam) influencing the measured amount or potency of morphine, use of neuromuscular blockers, hepatic or renal dysfunction, seriously compromised neurological status, or altered muscle tone. **Pretreatment:** Anesthetic management was standardized. Perioperative fluids were standardized to maintain a glucose infusion rate between 4 and 6 mg/kg/min; body temperature was kept within normal ranges. After the first arterial blood sample (baseline), patients received a second dose of 5 mcg/kg of fentanyl before surgical incision. At the end of surgery, all patients were given an intravenous loading dose of 100 mg/kg morphine.	
Interventions	**Intervention Characteristics** **Continuous infusion versus bolus administration** ***Dose regimen*:** Morphine continuous intravenous infusion 10 mcg/kg/h versus morphine every 3 hours after surgery 30 mcg/kg ***Description of procedure*:** The continuous morphine group started with a morphine infusion of 10 mcg/kg/h, combined with an intravenous placebo bolus (saline) every 3 h. The intermittent group received a continuous placebo infusion (saline), combined with an i.v. morphine bolus of 30 mcg/kg every 3 h. The first intermittent bolus (morphine or placebo) was given 3 h after surgery.	
Outcomes	*Pain assessed with validated methods during the administration of selected drugs* **Outcome type**: Continuous outcome Pain was assessed with the behavioral part of the COMFORT scale. In addition, the nurses completed an observational VAS for a clinical rating of pain in each child.	
Identification	**Sponsorship source:** Supported by the Dutch Organization for Scientific Research **Country:** The Netherlands **Setting:** Neonatal Intensive Care Unit **Comments:** None **Authors names:** Monique van Dijk, Nancy J. Bouwmeester, Hugo J. Duivenvoorden, Hans M. Koot, Dick Tibboel, Jan Passchier, Josien B. de Boer **Institution:** Department of Pediatric Surgery, Erasmus MC‐Sophia **Email:** vandijk@psys.azr.nl **Address:** Department of Pediatric Surgery, Erasmus MC‐Sophia, Rotterdam, The Netherlands	
Notes		
***Risk of bias***		
**Bias**	**Authors' judgement**	**Support for judgement**
Random sequence generation (selection bias)	Unclear risk	"Infants within these age categories were randomly assigned to CM or IM administration." "The hospital pharmacist prepared the study drugs; the randomization schedule was known to the pharmacist only and retained until the end of the trial." Not specified how the randomization schedule was generated
Allocation concealment (selection bias)	Low risk	"The hospital pharmacist prepared the study drugs; the randomization schedule was known to the pharmacist only and retained until the end of the trial".
Blinding of participants and personnel (performance bias) All outcomes	Low risk	"The hospital pharmacist prepared the study drugs; the randomization schedule was known to the pharmacist only and retained until the end of the trial." Blinded
Blinding of outcome assessment (detection bias) All outcomes	Low risk	"Pharmacist prepared the study drugs; the randomization schedule was known to the pharmacist only and retained until the end of the trial. Pain was assessed prior to surgery." Blinded
Incomplete outcome data (attrition bias) All outcomes	Low risk	"Excluded infants did not signiﬁcantly differ with regard to background characteristics, except age. This is primarily due to the low number of excluded neonates." "204 infants were allocated to this trial. However, five were lost to follow‐up; one infant died within the first hours after surgery due to irreversible pulmonary hypertension, three patients had a failing arterial line, and one patient experienced clinical signs of ventilatory depression. In addition, 18 patients were excluded because they did not comply with the inclusion criteria:"
Selective reporting (reporting bias)	Unclear risk	No registry reported so unable to compare to protocol
Other bias	Low risk	None

**Table:** Vaughn 1996

***Study characteristics***		
Methods	**Study design:** Double‐blind, randomized clinical trial **Study grouping:** Fentanyl continuous infusion versus bolus dosing every 2 hours	
Participants	**Baseline Characteristics** **Inclusion criteria:** Infants between 36 and 52 weeks postmenstrual age at the time of studies, who were admitted to the neonate intensive care unit. Patients underwent a surgical procedure after which at least 24 hours of narcotic analgesia was likely to be needed. **Exclusion criteria:** Patients that required assisted ventilation before surgery were not eligible. **Pretreatment:** None	
Interventions	**Intervention Characteristics** **Continuous infusion versus bolus administration** ***Dose regimen*:** Fentanyl by continuous infusion or bolus dosing every 2 hours ***Description of procedure*:** In the initial phase of the study, enrolled patients were randomly assigned to receive fentanyl by either continuous infusion beginning at 1 mcg/kg/h, or bolus dosing with a starting dose of 2 mcg/kg every 2 hours, thus providing equivalent cumulative dosing over time.	
Outcomes	*Pain assessed with validated methods during the administration of selected drugs* **Outcome type**: Continuous outcome After initiating the study medications, an observational pain assessment was performed. The observational pain assessment tool used was an adaptation of the Infant Pain Scale (IPS). *Duration of mechanical ventilation (days)* **Outcome type**: Continuous outcome Time to extubation	
Identification	**Sponsorship source:** Supported by the General Clinical Research Centers Program, National Center for Research Resources (NIH) **Country:** United States of America **Setting:** Neonate Intensive Care Unit **Comments:** None **Authors names:** Philip R. Vaughn, Susan F. Townsend, Elizabeth H. Thilo, Shirley McKenzie, Susan Moreland, Kerry Kawato **Institution:** Department of Pediatrics, University of Colorado School of Medicine and Department of Pharmacy, The Children's Hospital **Email:** Not provided **Address:** Pediatrics, University of Colorado Health Sciences Center, 4200 E Ninth Ave, BOX B‐195, Denver, CO, 80262	
Notes		
***Risk of bias***		
**Bias**	**Authors' judgement**	**Support for judgement**
Random sequence generation (selection bias)	Unclear risk	"Enrolled patients were randomly assigned to receive fentanyl by either continuous infusion (C) beginning at 1 pg/kg/h, or bolus dosing (B) with a starting dose of 2 kg every 2 hours, thus providing equivalent cumulative dosing over time." Not specified
Allocation concealment (selection bias)	Unclear risk	Not described in paper
Blinding of participants and personnel (performance bias) All outcomes	Low risk	"Caregivers were unaware of the patient’s group assignment (i.e. the caregivers did not know which of the two syringes contained the fentanyl)." Blinded
Blinding of outcome assessment (detection bias) All outcomes	Unclear risk	"Any intervention to support the patient was recorded by the bedside nurse. Apnea was determined by impedance pneumography and direct observation." Not stated whether these nurses were blinded
Incomplete outcome data (attrition bias) All outcomes	Low risk	"A description of the patients enrolled in both phases of the study is shown in Table 2." No concern
Selective reporting (reporting bias)	Unclear risk	No registry reported so unable to compare to protocol
Other bias	Unclear risk	"A significant limitation of the study is demonstrated by the larger proportion of patients who remained intubated and ventilated in phase 2 (6 of 13) compared with phase 1 (1 of 7). The study design did not incorporate a systematic approach to weaning ventilator support, and therefore interpretation of this observation is difficult." See quote

APS: Acute Pain Service CM: continuous morphine COI: continuous opioid infusion CS: COMFORT scale FLACC: Face, Legs, Activity, Cry, Consolability scale IM: intramuscular IPS: Infant Pain Scale i.v.: intravenous PNCA: parent‐ or nurse‐controlled analgesia VAS: visual analogue scale

### Characteristics of excluded studies [ordered by study ID]

**Table:**

Study	Reason for exclusion
Abiramalatha 2019	Wrong indication
Aguirre Corcoles 2003	Wrong patient population
Anand 1987a	Wrong study design
Anand 1987b	Wrong comparator
Anand 1992	Wrong intervention
Chiaretti 1997	Wrong patient population
Chiaretti 2000	Wrong patient population
ChiCTR‐IPR‐15006112	Wrong indication
Dake 1997	Wrong intervention
Gruber 2001	Wrong indication
Karl 2012	Wrong patient population
Kururattapun 1986	Wrong patient population
McEwan 2000	Wrong patient population
Michel 1995	Wrong patient population
NCT01094522	Wrong study design
Pan 2021	Wrong patient population
Waterworth 1974	Wrong patient population

### Characteristics of ongoing studies [ordered by study ID]

**Table:** NCT00004696

Study name	
Methods	Randomized clinical trial
Participants	Patients will be eligible if they are less than 12 months (part I) and less than 3 months (part II); had no hepatic or renal abnormalities; had no pulmonary disease causing baseline hypercarbia; had no pulmonary hypertension contraindicating use of 5% CO2 in rebreathing studies; had no allergies; and had no severe developmental delay that precludes analgesia scoring.
Interventions	Morphine postoperatively as intermittent intravenous bolus doses or as a continuous intravenous infusion targeted to reach a steady‐state concentration
Outcomes	Blood gases, continuous oximetry, and CO2 response curves and analgesia (using an infant pain score)
Starting date	
Contact information	
Notes	None

CO2: carbon dioxide

## Differences between protocol and review

The outcome "mechanical ventilation longer than 24 hours" was inserted a posteriori.

In the protocol, we specified the "COMFORTneo" pain scale, which was defined in 2009 (Van Dijk 2009); in the Results section, we included two studies which used the old version of the scale, i.e. "COMFORT", as they were published before 2009.
